# Intramuscular Electrical Stimulation Combined with Therapeutic Exercises in Patients with Shoulder Adhesive Capsulitis: A Randomised Controlled Trial

**DOI:** 10.29337/ijsp.25

**Published:** 2021-05-18

**Authors:** Sukumar Shanmugam, Lawrence Mathias, Nagarajan Manickaraj, K. U. Dhanesh Kumar, Praveen Kumar Kandakurti, Sathees Kumar Dorairaj, Ramprasad Muthukrishnan

**Affiliations:** 1 College of Health Sciences, Gulf Medical University, Ajman, United Arab Emirates; 2 K S Hegde Medical Academy, Nitte Deemed to be University, Mangaluru, India; 3 Menzies Health Institute Queensland, Griffith University, Queensland, Australia; 4 Nitte Institute of Physiotherapy, Nitte Deemed to be University, Mangaluru, India; 5 College of Health Sciences, Gulf Medical University, Ajman, United Arab Emirates

**Keywords:** Dry needling, Shoulder exercises, Electrical stimulation, Trigger points, Myofascial pain, shoulder pain, adhesive capsulitis, frozen shoulder

## Abstract

**Background:**

Myofascial trigger points (MTrPs) precipitate the shoulder pain severity and disability in patients with shoulder adhesive capsulitis (SAC). This study aims to compare the effectiveness of intramuscular electrical stimulation (IMES) combined with therapeutic exercises versus dry needling (DN) combined with therapeutic exercises in improving the clinical outcomes in patients with SAC.

**Methods and Materials:**

In this randomized controlled trial, IMES (n = 45) and DN (43) groups had received respectively IMES, and DN twice weekly for three consecutive weeks. Both groups received therapeutic exercises 1520 minutes, five days in a week during the second and third week. Pain, disability, kinesiophobia, number of active and latent MTrPs, shoulder abduction and external rotation range of motion were assessed at baseline, week-1, week-2, week-3 and follow-up at 3 months. A repeated measures ANOVA performed to find out the significant differences in the clinical outcomes between the groups.

**Results:**

The results of repeated measures of ANOVA shows that the post intervention timelines assessment scores of VAS, DASH, shoulder abduction and external rotation ROM, number of active and latent MTrPs and kinesiophobia were significantly (p. < 0.05) improved in both groups. However, IMES group had achieved a greater improvement over DN group (p. < 0.05) on the shoulder pain severity and disability, shoulder range of motion, number of active and latent MTrPs and kinesiophobia. Despite the significant statistical differences between the groups, IMES group did not achieve the minimal clinically important differences of 1.5cm and 11-points respectively for the VAS and DASH scores. No serious adverse effects occurred during the three weeks of treatment.

**Conclusion:**

IMES combined with therapeutic exercises is an effective treatment to reduce the shoulder pain severity and upper limb disability by deactivating the active and latent MTrPs and improving the shoulder abduction and external rotation range of motion in patients with SAC.

## 1. Introduction

Shoulder adhesive capsulitis (SAC) is one of the common painful conditions, where the patients develop pain and gradual decrease of active as well as passive range of motion [1]. The prevalence rate of SAC among the general population was estimated to be 25%, and adults aged between 30 to 50 years were commonly affected [2]. Pain intensity gradually reaches to the higher level after 3 months of symptoms onset [3], further it can limit upper limb functions [4].

Inflammation of the joint capsule and/or periarticular structures has a greater role for the occurrence of shoulder pain, especially during the acute stage SAC [5]. In later stages, myofascial trigger points (MTrPs) formation is important factor to contribute to the shoulder pain severity, soft tissue tightness and reduced range of motion [6,8]. The presence of active and latent-MTrPs in the shoulder girdle muscles found to be associated with fear avoidance belief behaviour during upper limb functions in individuals with painful shoulder disorders [7].

An MTrP is a hyperirritable spot within the discrete taut bands of the skeletal muscle fibers, which can develop sensory, motor, and autonomic dysfunctions [9]. The primary clinical manifestations of active-MTrPs are spontaneous referred pain, local twitch response, increased pain sensitivity whereas latent-MTrPs might be responsible for pain during pressure or muscle length changes in musculoskeletal pain disorders. However, both might increase the sensitization of pain receptors and alter the movement recruitment pattern [10]. Over the referral zone of active-MTrP, the muscles may develop latent-MTrPs [11].

According to previous study, increased MTrPs activity in the shoulder region might stimulate the sensitivity of MTrPs in the remote muscles; lead to development of widespread pain in patients diagnosed with myofascial pain syndrome (MPS) of shoulder [12]. Although the features of altered pain sensitivity within the MTrPs area confirm the peripheral source of nociception, the presence of widespread pain indicates the central sensitization of MTrP [13,14]. Overall, the optimal management for the deactivation of MTrP might reverse the sensitization process of pain, thereby attenuate the central and peripheral sensitization in musculoskeletal conditions [15].

The signs and symptoms of SAC with or without MTrPs were commonly managed by non-steroidal anti-inflammatory drugs, joint mobilization, muscle stretching, range of motion exercises and electrophysical agents in the regular clinical practice [16,17]. However, the clinical efficacy of pharmacological and conventional physiotherapeutic intervention was limited for the management of SAC associated with MTrPs [18]. Moreover, conventional therapies were found to be less effective for the MTrPs deactivation especially in patients with SAC.

It is pertinent to identify and treat the active and latent MTrPs by appropriate examination and treatment methods in case of painful shoulder disorders. In recent years, Dry needling (DN) and intramuscular electrical stimulation (IMES) were shown to be effective management of MTrPs in the wide range of musculoskeletal conditions [19]. However, there was no substantial evidence to support the clinical efficacy of IMES in patients diagnosed with SAC and presence of active and latent MTrPs.

DN is one of the standard interventions, where a thin solid acupuncture needle inserted into the muscles to deactivate the MTrPs to achieve pain relief and muscle relaxation [9]. DN has been applied as a standalone treatment or adjunct with other conventional physical and pharmacological therapies [20,21]. According to Brennan et al (2017), dry needling was effective as corticosteroid injection on reducing pain in patients with capsulitis associated hip pain [22]. Dry needling in an area of MTrPs and cervico-thoracic spinal segments i.e. paraspinal dry needling has been recommended to improve the clinical outcome of shoulder dysfunctions [23].

IMES is a minimally invasive electrotherapeutic method used to deliver electrical impulses into the muscles for achieving therapeutic benefits such as pain relief, movement reeducation and relaxation [24]. IMES through dry needles was expected to desensitize the peripheral and central pain mechanisms of MTrP. The studies of IMES on nonspecific thoracic pain [25], hemiplegic shoulder [26], and non-traumatic shoulder conditions [8,27] have been demonstrated a significant change in the pain score. Particularly, Chae et al (2005) reports that more than 40% reduction of pain following the IMES compared to the controlled intervention in patients with hemiplegic shoulder, which address therapeutic efficacy of IMES [26].

However, current evidence of IMES on pain and functional disability was limited for SAC [8]. Active and latent MTrPs deactivation might bring effective treatment that features with widespread pain associated with number of MTrPs, severity of pain, restricted joint movements and fear avoidance belief behaviour in individuals with SAC.

### 1.1 Study hypothesis and objectives

we hypothesized that IMES combined with therapeutic exercises may be more effective than DN combined with therapeutic exercises in improving the clinical outcomes in individuals with SAC.

1. Primary objective was to study the effectiveness of IMES combined with therapeutic exercises on shoulder pain and disability over dry needling combined with therapeutic exercises in individuals with SAC
2. Secondary objective was to study the effectiveness of IMES combined with therapeutic exercises on active range of motion, kinesiophobia and number of active and latent MTrPs over dry needling combined with therapeutic exercises in individuals with SAC.

## 2. Methods and materials

### 2.1 Study design and setting

This study was an assessor blinded randomized controlled trial and approved by the institutional ethics committee. The Helsinki convention norms for the research in human subjects were followed [28].

### 2.2 Participants selection criteria

Orthopedic surgeon (LM) recruited the potential participants with SAC and were screened further by the physiotherapists based on the inclusion and exclusion criteria. Inclusion criteria for this study includes, 1) participants aged between 3060 years irrespective of gender, 2) duration of adhesive capsulitis from >1-month to <4-months, 2) presence of MTrPs in the shoulder girdle muscles, 3) overall shoulder pain score of 3-cm in 0 to10-cm VAS, and a minimum availability of 60 to 120 degrees of active/passive shoulder abduction range of movement. The presence of hypersensitive knots with localized or diffused pain, taut band with or without referred pain identified by palpation to confirm the MTrPs

The exclusion criteria of this study follows: history of upper extremity fractures, dislocation, acute soft tissues rupture of the ipsilateral upper limb, diabetes mellitus, shoulder manipulation under local or general anesthesia, previous trigger point injection therapies such as botulism toxin and platelet rich plasma injection. The exclusion criteria also include participants with a history of systemic inflammatory conditions, needle phobia, nutritional deficiencies, hypothyroidism, pregnancy, anticoagulation therapy with bleeding disorders, hemiplegia, cervical intervertebral disc protrusion or any space occupying lesions affecting cervical spine, thoracic outlet syndrome, neuropathies, and presence of cardiac pacemakers.

### 2.3 Sample size

A priori sample size was calculated based on pain severity using minimal clinical important differences (MCID). The sample size estimations were performed using formula: n = 2(Z_ +_ Z)^2^ s^2^ / d^2^ [29] to achieve a larger between group effect size with 95% of confidence interval and 90% power at the 0.05 significance level. It was estimated from the sample size calculation that 76 participants (38 in each group) would meet the requirement to achieve the between-group minimum clinically important difference of 1.5 cm (2 cm) points from the baseline VAS score [30]. Considering the chances of 15% dropout rates, we decided to recruit a minimum of 90 participants (n = 45 in each group) to achieve the clinically meaningful between-group differences in the pain outcomes.

### 2.4 Randomization

In the present study, the participants were allocated into IMES and DN groups by the random number generation method. Block randomization was used for group allocation with each block containing of 6 random numbers for 15 blocks with the total sample size of 90 participants. All the blocks were concealed within a single large opaque envelope which was sent to the research supervisor and the outcome assessor was (DK) blinded.

### 2.5 Interventions

#### 2.5.1 Preparation for intervention

Prior to the IMES or DN, the participants were positioned in a comfortable prone lying position, and the skin over the cervical spine and trigger points areas of shoulder girdle muscles cleansed with antiseptic liquid. The location of MTrP and the spinous processs tip are marked from third to sixth cervical vertebra. Subsequently, a spot 1-cm lateral to the tip of the spinous process was identified and marked for the paraspinal dry needling [31], as these cervical segments innervated the shoulder muscles. All participants were informed about the possible experience of pinpricking and/or muscle twitch sensation, which would occur during IMES or DN [23].

#### 2.5.2 Dry needling for the Group I

##### Paraspinal dry needling

**Figure 1:**
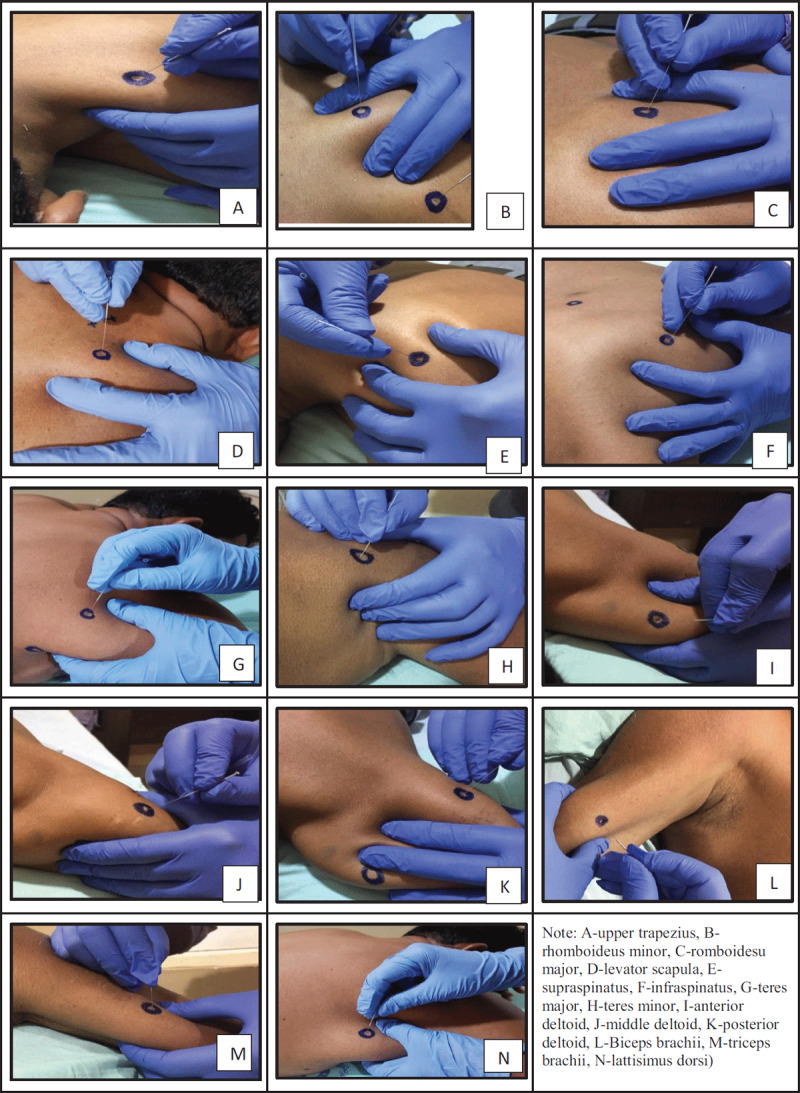
Dry needling procedure for shoulder girdle muscles.

The participants received DN for the identified active and latent MTrPs of the shoulder girdle muscles, and paraspinal segments (C3-C6) associated with the MTrPs of the shoulder muscles. We performed paraspinal DN prior to the dry needling of MTrPs by inserting the sterilized disposable acupuncture needle (30-mm thick 25-mm length, Cloud & Dragon, China) into the skin identified over the paraspinal region, approximately 1-cm lateral to the supraspinous line. In the inferomedial direction, the needles were penetrated deep into the cervical multifidi muscles until the needle touches the posterior laminar surface of the vertebrae [8,31] (***Figure 1***). This method of needling averts needle penetration into the vertebral artery, vertebral foramen and/or spinal cord.

##### Trigger point dry needling

DN for the MTrPs was performed over the identified trigger points locations at a suitable angle. The needles of suitable length (25-mm, 30-mm, 40-mm, 50-mm; Cloud & Dragon, China) and thickness (30-mm), depending on the depth of the MTrPs location inserted into the shoulder girdle muscles to deactivate the active and latent MTrPs. Subsequently, the inserted needles moved to-and-fro direction to elicit the local twitch responses [32,33], which further reaffirms the ideal placement of needle into the MTrPs. After the twitch response obtained, the dry needles were kept within the muscles approximately for 10 minutes to induce the mechanoreceptor mediated opioid release and muscle relaxation. The DN sessions were carried out twice a week for three weeks with a minimum of 4872 hours interval between the two consecutive needling sessions [8,23,33].

#### 2.5.3 Intramuscular electrical stimulation for Group-II

**Figure 2:**
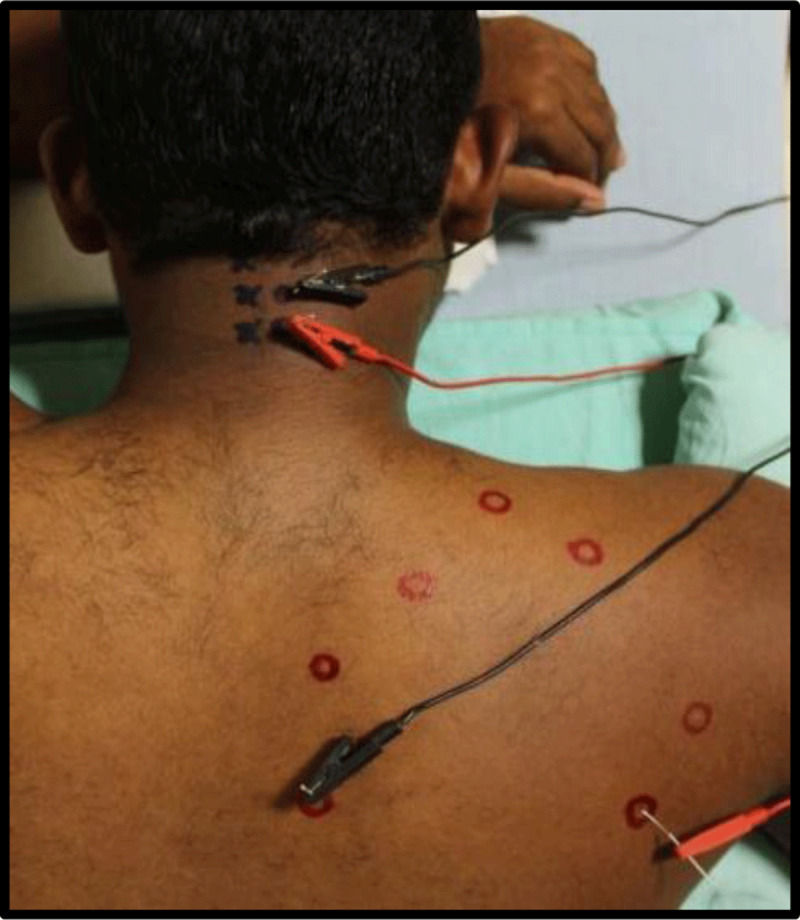
Procedure of intramuscular electrical stimulation. *Note*: Above picture shows the electrode placement for the IMES of Rhomboid and infraspinatus muscle. Black color lead indicate channel 1 and red color lead indicate channel 2. Cathode lead connected to the paraspinal level needle electrode and anode lead connected to the needle electrode of shoulder girdle muscles.

The participants received a similar method of dry needling in the MTrPs areas of shoulder muscles and paraspinal region of cervical spine. In addition, intramuscular electrical stimulation using inverse electrode placement was employed, where anode pole of the stimulator connected the needles of the MTrPs of the shoulder muscles and the cathode pole connected to the needles of the paraspinal region [8] using the alligator clip connector (***Figure 2***). The special programmed Vectrostim-100 (Technomed Electronics, Chennai, India), a double channeled multipurpose electrical stimulator was used to deliver electrical impulses with following parameters; pulse duration of 250-sec, pulse interval of 3 seconds (1 pulse per 4 seconds) with the stimulus intensity as tolerable by individuals. Each muscle was stimulated for 23 minutes (3045 muscle twitches) with the tolerable intensity twice a week for three consecutive weeks [8].

#### 2.5.4 Therapeutic exercises for both groups

Gentle muscle stretching was performed to maintain range of motion after the DN and IMES respectively in the group I and II during the first week. During the second and third weeks, the participants performed the active free movements through forward-backward, side-side, and clock-counterclock directions, 2030 repetitions in each direction, approximately lasting for 5 minutes [34]. Shoulder strengthening exercises was performed within the pain free range of shoulder movements using elastic thera-band, 10 repetition for each movement, twice daily, six days per week for three consecutive weeks [34,35].

### 2.6 Outcome measures

#### 2.6.1 Primary outcome

The severity of shoulder pain was assessed using visual analog scale (VAS: 0-10cm, where 0 indicates no pain and 10 indicates severe pain) which has a high intra and inter-rater reliability to measures of pain severity [36]. The functional disability was assessed using Disabilities of Arm Shoulder and Hand questionnaire (DASH: 0-100, where 0 indicates no disability and 100 indicates severe disability) which has good psychometric properties to measure the upper limb function following the various shoulder pain disorders [37].

#### 2.6.2 Secondary outcomes

Shoulder joint range of motion was measured using universal handheld goniometer [38,39] and Tampa Scale of Kinesiophobia (TSK-11) used to measure the movement avoidance behavior that might occur due to fear of pain (i.e. kinesiophobia). TSK-11 consists of 11 items, each items score ranges from 1 to 4 and therefore the total scores ranges from 11 to 44, where 11 indicates absence of kinesiophobia and 44 indicates severe kinesiophobia [40]. We assessed the VAS and DASH score, shoulder range of motion at the end of each week for three consecutive weeks, and then at 3 months follow-up. The number of active and latent MTrPs were assessed using manual palpation [41] and documented at baseline, 3-weeks post intervention and at 3-months follow-up period.

### 2.7 Statistical methods

The baseline demographic and clinical characteristics of participants were compared using chi-square test (for categorical data) and independent t-test (for qualitative and quantitative data) to assess the similarity between the groups. All the between-group comparisons for the primary and secondary outcomes performed based on the intention-to-treat analysis, using IBM SPSS version 21 (IBM, Chicago, IL). Linear regression analysis was employed to identify the potential covariates which had influence over the post intervention timeline outcomes of VAS, DASH, TSK-11, shoulder abduction and external rotation ROM, number of active and latent MTrPs. The between-group differences in the change score of primary and secondary outcome measures was analyzed using multivariate test; for which we used intervention groups (Group I and Group II) as independent variables, timeline change scores (1^st^ week, 2^nd^ week, 3^rd^, 3 months) as dependent variables, potential baseline scores as covariate. The significant between group differences based on the adjusted mean differences at 1^st^ week, 2^nd^ week, 3^rd^ week and 3 months were reported at 5% alpha level. Clinical significance was interpreted based on the effect size (partial eta squared: _p_^2^), standard mean differences (SMD) and confidence interval for the difference (CID) that meet the MCID of the primary and secondary outcomes.

## 3. Results

### 3.1 Recruitment

**Figure 3:**
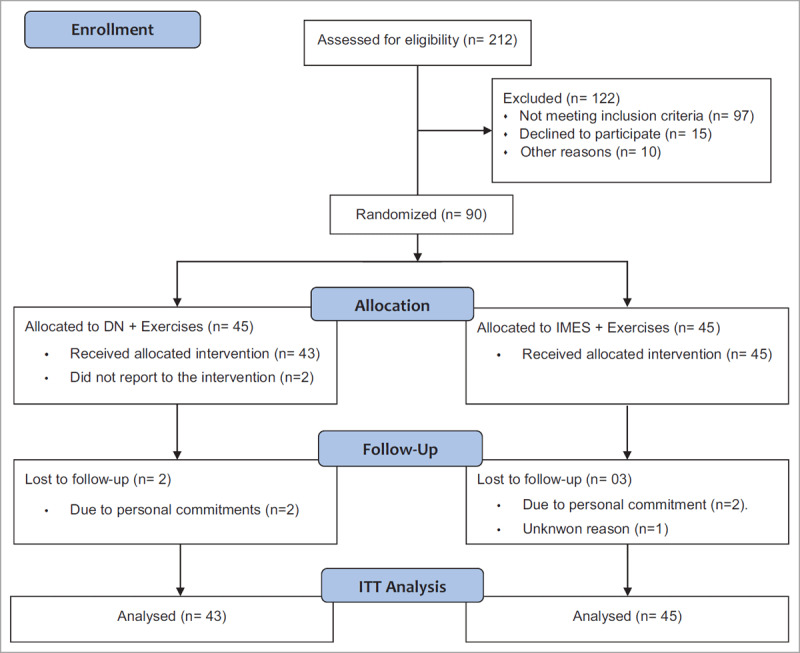
Consort flow diagram of study procedure.

Consort flow chart of study procedures shown in ***Figure 3***. For this study, 212 patients were screened and enrolled 90 eligible participants (54 female, mean age = 46.54 5.85 years). According to the collected information, 58% of participants reported with left SAC and diabetes mellitus with or without any shoulder pain disorders (n = 74) was the primary reason for the participants exclusion compared to the systemic illness (n = 21), trauma (n = 12), neurological (n = 8) and miscellaneous conditions (n = 7). After group allocation, 2 participants from DN group did not receive intervention due to the personal reasons, and collectively 88 participants (43 in DN, 45 in IMES group) completed the 3 weeks of interventions and considered for ITTA

### 3.2 Comparison of baseline outcome variables

The between groups comparison of baseline outcomes using Independent t-test showed the homogeneity in the duration of shoulder pain (p = 0.580), the number of active (p = 0.125) and latent-MTrPs (p = 0.370), VAS (p = 0.108), DASH (p = 0.547), TSK-11 (p = 0.213) and shoulder abduction (p = 0.294) and external rotation (p = 0.095) ROM. Similarly, chi-square test results revealed that number of participants with side of SAC (p = 0.124) and hand dominance (p = 0.263), use of medications (p = 0.124) was also similar at baseline. But, participants age (p = 0.032), and number of male and female participants (p = 001) were significantly different between the groups (***Table 1***).

**Table 1:** Baseline characteristics of study participants.


VARIABLES	DN + THERAPEUTIC EXERCISE GROUP	IMES + THERAPEUTIC EXERCISE GROUP

Age in years: Mean (SD)	45.26 (4.81)	47.82 (6.13)*

Gender: Male/Female	25/18	11/34*

Side affected: Right/Left	15/28	23/22

Hand dominance: Right/Left	41/02	40/05

Medication use: Yes/No	21/22	19/26

Duration of condition in Weeks: Mean (SD)	11.23 (3.26)	10.91 (2.05)

Number of active MTrPs: Mean (SD)	5.86 (1.10)	5.51 (1.01)

Number of latent LTrPs: Mean (SD)	6.47 (1.45)	6.20 (1.31)

NPRS score: Mean (SD)	7.47 (0.70)	7.18 (0.94)

TSK score: Mean (SD)	33.53 (4.31)	32.38 (4.33)

DASH score: Mean (SD)	62.86 (8.64)	61.72 (9.10)

Shoulder abduction ROM: Mean (SD)	83.05 (9.54)	85.38 (11.09)

Shoulder external rotation: Mean (SD)	35.88 (5.92)	37.87 (5.07)


DASH: disabilities of arm, shoulder and hand questionnaire, NPRS: numerical pain rating scale, TSK: tampa scale of kinesiophobia, IMES: intramuscular electrical stimulation, DN: dry needling, SD: standard deviation, * The Difference is significant at the .05 level.

### 3.3 Potential covariates

The linear regression analysis results (adjusted R^2^-value) showed that post intervention shoulder pain severity scores significantly predicted by the baseline VAS and number of active-MTrPs. Whereas, baseline DASH, TSK-11, shoulder range of motion and number of latent-MTrPs significantly predicted the post intervention clinical outcomes of upper limb disability and shoulder abduction and external rotation ROM.

### 3.4 Within groups difference in the clinical outcomes

The results of repeated measures of ANOVA shows that the post intervention timelines assessment scores of VAS, DASH, shoulder abduction and external rotation ROM were significantly improved from the respective baseline scores among participants received IMES or DN, combined with therapeutic exercises (***Figure 3***). Similarly, the number of active and latent MTrPs and kinesiophobia at week 3 (post intervention) and month 3 (follow-up) were significantly reduced from the baseline in the DN and IMES groups (***Table 2***).

**Table 2:** Analysis of weekly progression of clinical outcomes in IMES and DN groups.


OUTCOME MEASURES	GROUP	MEAN (SD) SCORES OF OUTCOME MEASURES AT DIFFERENT TIMELINES			

		WEEK-1	WEEK-2	WEEK-3	MONTH-3

VAS	IMES	2.71 (0.59)	1.78 (0.47)	1.00 (0.56)	0.42 (0.50)

	DN	3.60 (0.54)	2.05 (0.43)	1.51 (0.51)	0.63 (0.54)

DASH	IMES	20.39 (5.75)	11.46 (2.32)	5.66 (1.99)	2.15 (1.64)

	DN	23.78 (3.80)	13.85 (2.67)	8.65 (2.22)	3.49 (2.07)

Shoulder Abduction	IMES	132.22 (12.51)	147.24 (7.50)	160.76 (6.33)	170.16 (4.30)

	DN	123.79 (11.16)	139.37 (10.49)	152.72 (7.88)	166.70 (4.43)

Shoulder Ext. Rotation	IMES	54.82 (5.27)	65.84 (4.72)	76.42 (4.85)	83.42 (3.63)

	DN	49.53 (5.56)	60.88 (5.44)	72.70 (6.07)	80.88 (4.82)

TSK-11	IMES			14.95 (1.63)	11.16 (0.37)

	DN			16.60 (1.66)	11.51 (0.83)

Active MTrPs	IMES			0.20 (0.40)	0.20 (0.46)

	DN			0.93 (0.70)	0.95 (0.69)

Latent MTrPs	IMES			0.36 (0.57)	1.16 (0.71)

	DN			0.88 (0.82)	1.72 (0.96)


IMES: intramuscular electrical stimulation, DN: dry needling, SD: standard deviation.

### 3.5 Between groups difference in the clinical outcomes

The results of the between groups comparisons of shoulder pain and disability, shoulder abduction and external rotation range of motion, kinesiophobia and active and latent MTrPs were shown in ***Table 3***.

**Table 3:** Comparison of mean improvement in the clinical outcome measures between the IMES and DN groups.


OUTCOME MEASURES	POST INTERVENTION TIMELINE									FOLLOW-UP	
	
	WEEK 1			WEEK 2			WEEK 3			MONTH 3	
			
	IMES	DN		IMES	DN		IMES	DN		IMES	DN

VAS	4.55*	3.77		5.51*	5.30		6.28*	5.85		6.85	6.73
DASH	41.89*	38.51		50.76*	48.48		56.55*	53.69		60.07*	58.86
Abduction	46.78*	40.81		61.95*	56.23		75.74*	69.29		85.19	83.21
External rotation	17.31*	13.27		28.39*	24.56		39.02*	36.32		46.11*	44.42
TSK-11							17.64*	16.70		21.47	21.76
Active MTrPs							5.41*	4.83^a^		5.40*	4.81
Latent MTrPs							5.90	5.52		5.09*	4.69


Based on estimated marginal means: *The mean difference is significant at the .05 level.

#### 3.5.1 Shoulder pain

Between the groups adjusted mean difference of the VAS score at week-1 (SMD = 0.77, CID = 0.57 to 0.97, p = 0.001, F = 60.962, _p_^2^ = 0.421), week-2 (SMD = 0.21, CID = 0.025 to 0.39, p = 0.026, F = 5.133, _p_^2^ = 0.058), week-3 (SMD = 0.43, CID = 0.22 to 0.65, p = 0.001, F = 16.242, _p_^2^ = 0.162) shows that the participants received IMES were significantly higher than the DN group. However, the mean difference (SMD = 0.12, CID = -0.08 to 0.32, p = 0.232, F = 1.448, _p_^2^ = 0.017) between the groups at 3-month follow-up was not significant.

#### 3.5.2 Shoulder disability

In case of shoulder disability (DASH score), the between groups adjusted mean difference at week-1 (SMD = 3.38, CID = 2.35 to 4.42, p = 0.001, F = 42.099, _p_^2^ = 0.339), week-2 (SMD = 2.28, CID = 1.49 to 3.07, p = 0.001, F = 33.048, _p_^2^ = 0.287), week-3 (SMD = 2.86, CID = 2.12 to 3.61, p = 0.001, F = 58.104, _p_^2^ = 0.415) and 3-months (SMD = 1.21, CID = 0.56 to 1.86, p = 0.001, F = 13.865, _p_^2^ = 0.145) show that the participants received IMES achieved higher clinical outcome compared to the DN in all the timelines.

#### 3.5.3 Shoulder Abduction

The between groups adjusted mean differences in the shoulder abduction ROM at week-1 (SMD = 5.97, CID = 8.00 3.93, p = 0.001, F = 34.094, _p_^2^ = 0.291), week-2 (SMD = 5.72, CID = 8.55 to 2.89, p = 0.001, F = 16.201, _p_^2^ = 0.163), week-3 (SMD = 6.45, CID = 9.15 to 3.75, p = 0.001, F = 22.635, _p_^2^ = 0.215) and month-3 (SMD = 1.98, CID = 0.17 to 4.14, p = 0.067, F = 3.362, _p_^2^ = 0.039) show that the participants received IMES achieved higher improvement compared to the DN group in all the timelines except 3rd month follow-up.

#### 3.5.4 Shoulder External rotation

Similarly, the between groups adjusted mean differences for the external rotation ROM at week-1 (SMD = 4.04, CID = 5.08 to 2.99, p = 0.001, F = 59.512, _p_^2^ = 0.421), week-2 (SMD = 3.83, CID = 5.17 to 2.51, p = 0.001, F = 32.927, _p_^2^ = 0.287), week-3 (SMD = 2.69, CID = 4.37 to 1.01, p = 0.002, F = 10.150, _p_^2^ = 0.110) and month-3 (SMD = 1.69, CID = 3.06 to 0.33, p = 0.016, F = 6.099, _p_^2^ = 0.069) show that the participants received IMES achieved higher improvement compared to the dry needling groups in all the timelines.

#### 3.5.5 Kinesiophobia

The multiple pairwise comparison results show that IMES was found to be more effective than the dry needling on the clinical outcome of the movement avoidance behavior of shoulder at 3-weeks post intervention (SMD = 0.94, CID = 0.06 to 1.81, p = 0.035, F = 4.567, _p_^2^ = 0.052). However, at 3 months follow-up no significant between the groups differences was observed (SMD = 0.29, CID = 1.36 to 0.77, p = 0.582, F = 0.305, _p_^2^ = 0.004).

#### 3.5.6 Number of active and latent MTrPs

The multiple pairwise comparison results show that IMES was found to be more effective than the dry needling on the clinical outcome of the number of active MTrPs at 3-weeks post intervention (SMD = 0.58, CID = 0.28 to 0.88, p = 0.001, F = 15.157, _p_^2^ = 0.151) and 3 months follow-up (SMD = 0.59, CID = 0.32 to 0.86, p = 0.001, F = 18.342, _p_^2^ = 0.177. But, in case of latent MTrPs a significant difference was not obtained at 3-weeks post intervention (SMD = 0.38, CID = 0.01 to 0.76, p = 0.054, F = 3.803, _p_^2^ = 0.043) and achieved significant difference at 3-month follow-up (SMD = 0.39, CID = 0.01 to 0.77, p = 0.043, F = 4.215, _p_^2^ = 0.047).

### 3.6 Adverse effects

According to the patients reports, adverse effects in the DN and IMES during the 3 weeks of treatment shown in ***Table 4***. Among the different adverse effects, post needling soreness was commonly reported by the participants. No serious adverse effects occurred during the three weeks of treatment.

**Table 4:** Adverse effects of DN and IMES in our study participants.


ADVERSE EFFECTS	NUMBER OF PARTICIPANTS EXPERIENCED ADVERSE EFFECTS		

	DN	IMES	TOTAL

Dry-needling induced Soreness	41	39	80

Severe pain during needling	2	3	5

Profuse sweating	4	5	9

Excessive post-needling pain	2	Nil	2


## 4. Discussion

This randomized clinical trial was undertaken based on the hypothesis that IMES combined with therapeutic exercises may deactivate the active and latent MTrPs of shoulder girdle muscles, thereby reducing pain, disability, movement avoidance behaviour and improve range of motion more effectively than DN combined with therapeutic exercises. Therefore, we aimed to find-out the between group differences based on the mean change scores (i.e. baseline - post intervention timelines). Collectively, the statistically significant differences between the groups interpreted based on the mean differences (and 95% confidence interval) that follow the minimal clinically important difference.

Study data showed a significant change in the post intervention and follow-up assessment scores of primary and secondary outcomes compared to the baseline (***Table 2***). The observed differences in the shoulder pain severity and upper limb disability scores at week-1, week-2, week-3 and month-3 (not for VAS) favoring IMES. While both the primary outcomes (VAS and DASH) show statistically significant differences between the groups, the mean differences did not meet the MCID values, i.e. 1.5 for pain, and 11-points for disability outcomes [29]. However, an important point to be noted here was that IMES achieved a greater improvement over DN in the shoulder pain severity (IMES vs DN: VAS Mean difference 0.77 CI = 0.57 to 0.97) at week 1.

The clinical efficacy of IMES has been evidenced in various musculoskeletal conditions including myofascial pain syndrome of shoulder [8,25]. However, only a few studies have documented IMES effects on SAC [8]. In this study, the theoretical and scientific concept was that the deactivation of MTrPs may reduce the widespread shoulder pain and improve the shoulder ROM. With this similar concepts, but using different stimulation parameters (Group I: 3Hz, 4-stimuli/site, Group II: 1Hz, 1-stimuli/site) Chu et al (2008) found significant pain relief and increased ROM by twitch obtaining IMES in patients with chronic myofascial pain syndrome of the shoulder [26]. Our current study used different stimulation parameters; 1-Hz stimuli for each MTrPs site for 3045 repeated contractions in 23 minutes. Interestingly, here we achieved similar improvement in pain and ROM outcome. According to our IMES method, all hyperirritable spots of muscles around the shoulder joint must be given equal importance to treat ACS effectively.

Once the patients pain intensity becomes mild or moderate after IMES, the clinician should apply therapeutic exercises to increase the ROM and pain relief. One of the studies in current literature supports that the application of IMES in addition to the therapeutic stretching can improve the pain reduction compared to standalone exercise interventions [42]. Among the couple of systematic reviews, one study suggest for supervised exercises in combination with electrotherapy [17], and another study recommend therapeutic exercises as a primary intervention [16] to produce significant reduction of pain and improvement in the shoulder range of motion in patients with adhesive capsulitis of shoulder. In our study, IMES was implemented as primary intervention and therapeutic exercises as adjunct therapy which also produced significant clinical outcomes as previous study findings.

Note that our study participants had achieved significant reduction of pain and functional improvements after receiving the standalone IMES or DN during the first week itself. Later, the supervised therapeutic exercises during the second and third week, and unsupervised home based exercises might have played a major role for the accelerated functional recovery and non-recurrence of pain symptoms during the 3 months intervention period.

On the other hand, a case series with 9 patients of nontraumatic shoulder pain disorders including ACS evidenced the short- and long-term therapeutic effectiveness on shoulder pain and disability post IMES using inverse electrode placement [8]. Using similar electrode placement, our current study employed a different type of electrical current parameters, which eventually produced almost similar clinical outcomes. Generally, the antidromic conduction of motor and orthodromic conduction of mechanoreceptive sensory pathways can facilitate neuromodulation of pain and movement function [43]. Since the active electrode was placed in the cervical spine of associated myotome/dermatome, the enhanced multidirectional conduction of neural pathways might be influenced the faster pain relief [8,43]. According to the findings of current and previous studies irrespective of the variations in the stimulation methods, it should be noted that IMES can be an effective and alternative method for SAC management [8].

The effects of IMES on painful conditions such as low back pain [44], non-specific thoracic pain [25] and hemiplegic shoulder [26] has been evidenced in the past years. As example, IMES through needle electrodes placed over the upper thoracic paraspinal region have produced a 23-mm change in the 0100-mm VAS in patients with non-specific thoracic spine pain [25]. Most importantly, a randomized controlled trial on effects of IMES in hemiplegic shoulder pain with or without shoulder subluxation exhibited more than 40% reduction of pain symptoms compared to the controlled intervention [26]. As a general treatment approach to MPS, a randomized controlled study states that IMES in addition to the stretching exercises improve the pain reduction compared to standalone exercise intervention [42]. Thus, the clinical efficacy of IMES was substantial in the wide range of musculoskeletal conditions; most importantly current study findings can be reproducible in other painful musculoskeletal disorders associated with MTrPs.

Secondarily, this study compared the effect of DN and IMES on kinesiophobia, number of MTrPs and shoulder ROM. Literature suggest that the presence of active and/or latent MTrPs can sensitize peripheral and central nociceptive neurons which lead to the development of painful movements with fear avoidance behaviour. Therefore, our study employed IMES or DN to relax the tight muscles and to reduce the movement avoidance behaviour by deactivating both active and latent MTrPs. As a token of proof, our study interventions (i.e. IMES and DN) produced significant improvement on movement avoidance behaviour, number of active and latent-MTrPs and shoulder range of motion (abduction and external rotation) in patients with SAC. In fact, IMES has achieved better clinical outcomes than the DN. And the between group mean differences have met the MCID value of 3 for external rotation ROM at week-1, week-2 and week-3, but not for shoulder abduction range of motion (***Table 3***).

Dry needling is also one of the minimal invasive procedures used by clinicians to treat the painful conditions of shoulder [45]. Thus, DN with or without IMES can be an alternative method to relieve pain and associated functional disabilities in patients with adhesive capsulitis of shoulder [46]. In case of a patients unwillingness to take medications, a decision can be made to employ IMES to treat pain and disability associated with MTrPs if the patient is ready to sign consent for the treatment [23].

## 5. Conclusion

Standalone IMES and DN significantly reduced the shoulder pain, disability, range of motion at week-1. However, IMES was more effective than DN for pain relief, MTrPs deactivation and improving the external rotation range of motion. Based on the results, IMES and DN in combination with therapeutic exercise produced better clinical outcomes at week-2, week-3 weeks (post intervention) and month-3 (follow-up). Henceforth, we suggest IMES or DN in addition to therapeutic exercises to manage the signs and symptoms of SAC during regular clinical practice. IMES and DN treatments have not produced serious adverse events during this trial; however, cautions should be taken while performing it as a safe clinical practice.

## 6. Study strength

This is a first randomized controlled trial comparing the effectiveness of IMES over DN and combined with therapeutic exercises in individuals with SAC (symptoms duration: >4 weeks and <16 weeks) associated with MTrPs. In this study, we used a unique electrical stimulator with custom made stimulation parameters specially designed for producing the safe and smooth muscle contraction to deactivate the MTrPs. Most importantly, our study emphasized on simultaneous stimulation of shoulder as well as cervical paraspinal muscles by appropriately placing the needle electrodes. This method of stimulation effectively desensitizes both peripheral and central nociceptive neurons instead of acting only on the peripheral muscles.

According to the post intervention survey, the patients satisfaction rate was higher for the IMES stimulation method. None of the patients have discontinued the intervention during 3 weeks, which indicates its clinical safety, and feasibility to apply in patients with painful shoulder disorders. Standalone dry needling or IMES was employed during the first week of SAC management which identified the significant differences in the primary outcome measures. And more than 50% of reduction in shoulder pain severity was observed within a week of IMES treatment.

## 7. Study limitations and future recommendation

One of the limitations of our study was that neither a typical control group nor placebo-needling (and placebo IMES) included. Thus, the exact values of clinical significance for DN or IMES treatment (against the control group) could not be identified based on the MCID. Secondly: since the patients were positioned in prone lying, IMES was not delivered into the MTrPs of pectoralis minor and proximal pectoralis major muscles due to the technical difficulties. Hence future studies can compare the IMES over typical control groups with only pharmacological interventions. This study excluded patients of both controlled and uncontrolled diabetes mellitus. Since the prevalence of adhesive capsulitis of the shoulder is high among the diabetic patients, at least the patients with controlled type-I diabetes mellitus may be tried for IMES or DN by modifying the frequency and intensity of treatments to promote faster clinical recovery.
